# Re-examining electrophysiological evidence for proactive suppression of salient visual distractors

**DOI:** 10.3758/s13414-025-03180-w

**Published:** 2025-11-26

**Authors:** John J. McDonald, Daniel Tay, Rebecca Carson

**Affiliations:** Department of Psychology, Simon Fraser University, 8888 University Drive, Burnaby, BC V5A 1S6 Canada

**Keywords:** Visual search, Distraction, Salience, Suppression, Distractor positivity (P_D_)

## Abstract

Salient-but-irrelevant color singletons often elicit a positive component in the event-related potential (the P_D_) rather than a negative component associated with attentional selection (the N2pc). The positivity is often assumed to reflect inhibitory control processes that prevent salience-driven distraction, particularly when the positivity emerges before the time range of the N2pc. To be certain that this “early P_D_” is associated with inhibition, it is necessary to show that the positivity is absent when participants search for the color singleton. Here, we replicated a seminal letter-search task in which a singleton distractor was found to elicit an early positivity (Experiment 1) and then instructed participants to detect the presence of the same singleton (Experiment 2). We discovered that the early positivity is present both when participants ignored the singleton and when they searched for the singleton. These results suggest that the early positivity is associated with salience processing rather than inhibition that prevents distraction.

## Introduction

The signal suppression hypothesis was introduced to explain how individuals manage to ignore salient visual distractors under certain conditions (Sawaki & Luck, 2010). The hypothesis is that salient visual stimuli, such as color singletons, automatically generate “attend-to-me” signals (herein called priority signals) that will attract attention to locations of these stimuli unless the priority signals are suppressed before attention can be pulled away from its current spatial focus. This idea is similar to other attention-control theories in proposing that people can ignore salient-but-irrelevant visual items, but it is unique in proposing that suppression is the only way to prevent attention capture by salient distractors. Earlier theories, including the contingent capture theory and the dimension-weighting account, proposed that distraction can be prevented by upweighting the target’s features (Folk & Remington, 1998; Folk et al., 1992; or dimension: Found & Müller, 1996) or by down-weighting (i.e., suppressing) the distractor’s features (Liesefeld & Müller, 2019; Luck et al., 2021).^1^ Proponents of the signal suppression hypothesis have asserted that distractor suppression is possible when a person uses a feature-based strategy to find a target (e.g., “find the circle”) but not when they use a salience-based strategy (e.g., “find the singleton”), presumably because the latter strategy makes it difficult to distinguish between target and distractor (Gaspelin et al., 2015; Sawaki & Luck, 2010; see also Bacon & Egeth, 1994).

Behavioral results from two paradigms have provided support for the signal suppression hypothesis. In one paradigm, participants were asked to discriminate the orientation of a line inside a target shape that appeared in a circular array of heterogenous shapes (Gaspelin et al., 2017). The target and all but one of the nontarget shapes were filled with the same color, but on some trials one shape was filled with a different color (the singleton distractor). Due to the short length of the line, participants had to move their eyes toward the target shape to perform the task. Most of the first saccades were directed at the target shape, but some landed on one of the target-colored distractors. Critically, fewer first saccades landed on the singleton distractor than on any other distractor. The relative paucity of singleton-directed saccades indicates that the singleton may have been suppressed to prevent it from capturing attention. In another paradigm, participants discriminated the positioning of the line within the target shape without making saccades (Gaspelin et al., 2015). Some of the search trials were replaced by letter-probe trials, on which letters appeared briefly inside the shapes. Fewer correctly recalled letters were positioned on the singleton distractor than on one of the target-colored distractors, indicating once again that the singleton may have been suppressed.

Although the results from the oculomotor capture and probe-recall paradigms are consistent with the signal suppression account, they are also consistent with feature-upweighting accounts (Gaspelin et al., 2015, 2017; Oxner et al., 2023). For example, when participants search for a green target, processing of green distractors may be upweighted relative to red distractors. Such display-wide feature enhancement occurs at the neural level (in visual area V4) when monkeys search for target items with prespecified colors (Bichot et al., 2005). Thus, the relative paucity of singleton-directed saccades may have reflected enhancement of the nonsingleton distractors rather than suppression of the singleton distractor. In line with this feature-enhancement interpretation, the relative reduction in recalling singleton-location letters on probe-recall trials is not evident when the colors of the target and nonsingleton distractors do not match (Oxner et al., 2023, 2024a, b).

Event-related potentials (ERPs) have been used to help determine whether salient distractors can be suppressed during visual search (e.g., Gaspar & McDonald, 2014; Sawaki & Luck, 2010). Singleton distractors that capture attention elicit negative ERP activity over the posterior contralateral scalp, from approximately 200 ms until approximately 300 ms (i.e., in the time range of an N2 peak; Hickey et al., 2006; McDonald et al., 2013). This posterior contralateral N2 (N2pc) is hypothesized to reflect a spatial filtering process that enhances processing of the target relative to surrounding items (Eimer, 1996; Hickey et al., 2009; Luck & Hillyard, 1994; Tay et al., 2019). In contrast, singleton distractors that are suppressed may be expected to elicit positive ERP activity over the posterior contralateral scalp in the same time range as the N2pc (Gaspar & McDonald, 2014; Hickey et al., 2009). This contralateral positivity, which we called the distractor positivity (P_D_), is hypothesized to be associated with suppression of irrelevant visual items that may interfere with target processing (Hickey et al., 2009).

**Fig. 1:**
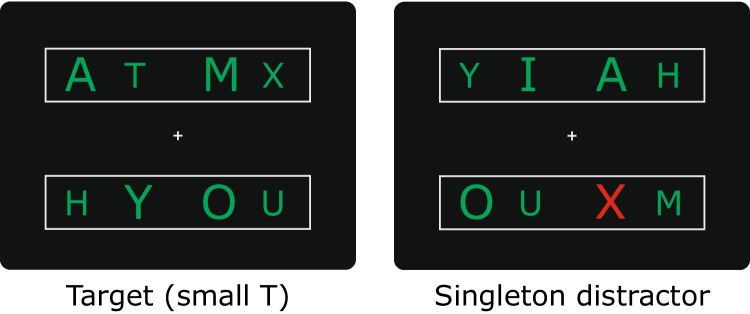
Example target and salient-distractor arrays used in Experiment 1. In Experiment 2, the color singleton served as the target. (Color figure online)

To test their signal suppression hypothesis, Sawaki and Luck (2010) recorded ERPs to letter arrays like the ones shown in Fig. 1. Approximately 71% of trials contained eight same-color letters (e.g., all green), while the remaining trials contained a color singleton (e.g., red letter) that was irrelevant to the task at hand. Participants were instructed to press a button when the array contained a specific target letter (e.g., a small, green T). As expected, targets and target-like distractors (e.g., a large green T) were found to elicit the N2pc, indicating that participants had to attend to both letters to determine if they were targets or not. In contrast, the irrelevant color singleton was found to elicit a contralateral positivity beginning in the time range of the earliest P1 peak (~100–120 ms after stimulus onset) over the posterior scalp. Sawaki and Luck interpreted this positivity as a P_D_ and concluded that the salient-but-irrelevant singleton was suppressed.

Sawaki and Luck’s (2010) study showed definitively that singleton distractors do not invariably elicit the N2pc. This finding, which has been replicated in a variety of different search tasks (e.g., Jannati et al., 2013), indicates that people can ignore salient items when they do not resemble the target. However, the evidence for distractor suppression is less clear cut, because attention and other factors may also boost contralateral positive voltages starting in the time range of the P1. For example, when attention is cued in advance to one side of a bilateral letter array, the ERP elicited by the array is more positive contralateral to the attended side than ipsilateral to the attended side, starting in the time range of the P1 (Heinze et al., 1990; Luck et al., 1990; for similar results using nonletter stimuli, see Fukuda & Vogel, 2009; Hoffmeister et al., 2022; Livingstone et al., 2017; McDonald et al., 2005; Störmer et al., 2009). The salience of a visual singleton appears to trigger a similar early contralateral positivity independent of whether the item is eventually attended or ignored (Corriveau et al., 2012; Fortier-Gauthier et al., 2012; Jannati et al. 2013). Thus, it is unknown whether the contralateral positivity reported by Sawaki and Luck (2010) reflects the suppression of a salience-driven priority signal or the salience-driven priority signal itself.

Sawaki and Luck’s (2010) seminal study included a control experiment to rule out a purely sensory account of the contralateral positivity. In it, four small squares were added to the letter arrays, within 0.75º of fixation. Participants were instructed to detect a specific square (e.g., with a gap on top) that occurred on 50% of the trials. The peripheral letters appeared at the same time as the central, four-item search array but were completely task irrelevant. Sawaki and Luck reasoned that “attention would be so focused and held at the central region by this highly perceptually demanding task that it would not be necessary to further suppress salient singletons at peripheral locations” (p. 1463). Consistent with this assumption, no contralateral positivity was observed in the control experiment. Sawaki and Luck concluded that the contralateral positivity did not reflect a purely bottom-up sensory process because it can be eliminated with a change in attentional instructions. However, the findings did not unequivocally link the contralateral positivity to suppression because the change in task may have eliminated the priority signal itself (and thus the need for suppression). Along these lines, Sawaki and Luck theorized that salient singletons might not trigger priority signals “when spatial attention is strongly focused on a different region of the display” (p. 1467).

The purpose of the present study was to determine whether the contralateral positivity reported by Sawaki and Luck (2010) reflects suppression of a singleton-driven priority signal or is more closely associated with the priority signal itself. We did this by first replicating their letter-search task, in which the color singleton was irrelevant (Experiment 1), and then asking a new sample of participants to detect the presence of the color singleton in the same randomly intermixed singleton-present and singleton-absent letter arrays (Experiment 2). Salience-driven priority signals should be roughly equivalent across the two experiments because participants had to monitor the same wide region of space for a specific target. If the contralateral positivity reflects suppression of a singleton-driven priority signal, it should be present only when participants attempt to ignore the color singleton. However, if the contralateral positivity is more closely associated with the priority signal itself, it should also be evident when participants search for the color singleton.

## Experiment 1

### Methods

The research ethics board at Simon Fraser University approved the research protocol used in this study. Data and materials are available upon request.

#### Participants

Sample size was determined a priori to have sufficient power (1 − β =.80) to detect the presence of medium-sized N2pc and P_D_ components (Cohen’s *d*_z_ = 0.50) by one-tailed, one-sample *t* test (see electrophysiological recording and analysis section for rationale of one-tailed tests). Small-amplitude ERP components like the contralateral positivity in Sawaki and Luck’s (2010) study are typically medium or large effects when the contralateral-ipsilateral difference waves are clean and the sample size is sufficient (Tay et al., 2019). Using G*Power, we determined that a sample size of 27 was required. For comparison, Sawaki and Luck (2010) ran 12 participants in each of their seminal experiments.

Thirty undergraduate students from Simon Fraser University participated after giving written informed consent. For their participation, each person earned either $20 or course credit as part of a departmental research participation system. All subjects reported normal or corrected-to-normal visual acuity and were tested for normal color vision using Ishihara color plates prior to participation. Data from three participants were excluded from further analyses because more than 30% of their trials were contaminated by ocular artifacts (rejection criterion set in advance). These participants were replaced to achieve our target sample size. Of the remaining 27 participants (mean age: 19.6 years), 16 were women and 22 were right-handed. No participant reported a history of neurological disorder.

#### Apparatus

The experiment was conducted in a sound-attenuated and electrically shielded chamber that was dimly lit by DC-powered LEDs. Visual stimuli were presented on a height-adjustable LCD monitor running at 120 Hz (Benq XL2420T). Participants sat in a chair and viewed the monitor at a distance of approximately 57 cm. Responses were made by pressing the green button of a Logitech gamepad with their right thumb. A Windows-based computed controlled stimulus presentation and registered participants’ button presses using Presentation (Neurobehavioral Systems, Inc., Albany, CA). A custom software (Acquire) recorded EEG from a second, Windows-based computer, which housed a 64-channel A-to-D board (PCI-6071e, National Instruments, Austin, TX) that connected to an EEG amplifier system with an input impedance of 1 GΩ (SA Instruments, San Diego, CA). The stimulus-control and EEG-acquisition computers were situated outside of the testing chamber.

#### Stimuli and procedure

Experiment 1 stimuli and procedure were based on Sawaki and Luck’s (2010) second experiment, in which participants detected target letters that could appear in either of two rectangles positioned above and below fixations. Stimuli were presented on a black background with continuously visible fixation cross (0.4º × 0.4º, 11.5 cd/m^2^) at the center of the display and two continuously visible gray rectangles (18.8º wide × 3.3º tall, 11.5 cd/m^2^) centered 4.3º above and below the fixation cross. Each stimulus array contained four letters in the upper rectangle and four letters in the lower rectangle (Fig. 1). Their positions were centered 4.3º above or below the horizontal meridian and 5º or 8º to the left or right of the vertical meridian. The letters were printed in upper-case Verdana font and were sampled without replacement from a set of nine letters (A, H, I, M, O, T, U, X, Y). Within each rectangle, two letters were small (0.7–1.6º wide × 1.9º tall) and two letters were large (1–2.3º × 2.4º). Letter arrays were presented for 200 ms, followed by a 600–700-ms inter-array interval (SOA = 800–900 ms). As detailed below, letters could be green (*x* = 0.31, *y* = 0.63; 19.7 cd/m^2^) or red (*x* = 0.65, *y* = 0.32; 17.8 cd/m^2^). Each participant performed 36 blocks of 56 trials after completing one or two practice blocks. Blocks were separated by participant-controlled rest periods that lasted for at least 10 s.

Participants were instructed to keep their eyes directed at the central cross and to remain still throughout each block of trials. They were also encouraged to comfortably reduce blinking during the task and to blink during rest periods. At the beginning of each block, one specific-sized letter (e.g., a small H) was designated to be the target. The target letter was selected randomly from an array of 36 letters (each of the 18 letter × size combinations was represented twice in the array). Participants were instructed to press the response button when the stimulus array contained a target at any one of the eight upper- or lower-field locations. Speed and accuracy were stressed equally.

The stimulus arrays contained eight nontarget letters printed in the same color on 288 trials (~14.3%). These standard letter arrays were green in half of the blocks and red in the other half (randomly intermixed across blocks). Within each block, three other types of stimulus arrays were randomly intermixed with standard arrays:

1. Target arrays, in which the target appeared in the standard letter color
2. target-like-distractor arrays, in which one of the nontarget letters had the same identity as the target (e.g., both H) but was different in size (e.g., large rather than small), and
3. singleton-distractor arrays, in which one of the nontarget letters appeared in the nonstandard color (red if the standard letters were green, or vice versa).

The identity of the singleton distractor was different from that of the target, but its size could be the same (determined randomly). Each of these three nonstandard display types appeared on 576 trials (~28.6%).

Responses within a 100–800-ms window were analyzed (as in Sawaki & Luck, 2010). Across all participants, an average of 2.2% of trials were discarded from the behavioral and ERP analyses because responses fell outside of this window.

#### Electrophysiological recording and analysis

Electroencephalograms (EEGs) were recorded from 25 Ag/AgCl electrodes positioned at Fp1, Fpz, Fp2, F7, F3, Fz, F4, F8, T7, C3, Cz, C4, T8, P7, P3, Pz, P4, P8, PO7, POz, PO8, O1, Oz, O2, and M1. The last electrode was fixed directly to the left mastoid, while the other 24 electrodes were mounted in an elastic cap (Sands Research). These 25 electrodes were referenced to a right-mastoid (M2) electrode during recording and were re-referenced offline to the average of the two mastoids. A separate bipolar channel was used to record the horizontal electrooculogram (HEOG) from Ag/AgCl electrodes positioned one centimeter lateral to the outer canthus of each eye. EEG and HEOG channels were amplified with a gain of 20,000 within a pass-band of 0.01–100 Hz (two-pole Butterworth filters) and were digitized at 500 Hz. A semi-automatic procedure was performed to remove epochs of EEG that were contaminated by horizontal eye movements, blinks (and vertical eye movements; monitored on Fp1), and amplifier blocking (for details, see Tay et al., 2022). An average of 14.2% of trials were excluded from the ERP analyses because of artifact contamination. The remaining trials were then used to create averaged ERP waveforms. The ERPs were digitally low-pass filtered (half-amplitude cutoff at 30 Hz) to remove high-frequency activity and digitally re-referenced to the average of the two mastoids. A 100-ms interval immediately preceding stimulus onset was used for baseline correction and all mean-amplitude measures.

ERPs time-locked to the onset of the letter arrays were computed separately for target arrays, target-like-distractor arrays, and singleton-distractor arrays. For each participant, the ERP waveforms were collapsed across left and right visual hemifields and left and right electrode sites to create waveforms recorded contralateral and ipsilateral to the lateral singleton in each array. Except where otherwise noted, trials with upper- and lower-field targets (or distractors) were combined to create the ERP waveforms. Lateralized difference waveforms were derived for each of the array types by subtracting the ipsilateral waveform from the corresponding contralateral waveform using lateral occipital electrode sites PO7 and PO8. These electrodes were selected a priori on the basis of numerous N2pc studies from our lab and elsewhere (e.g., Gaspar & McDonald, 2014; Tay et al., 2019). Positive voltages were plotted upward such that the N2pc and P_D_ would appear as downward and upward deflections, respectively.

All N2pc and P_D_ measurements were taken from the contralateral-ipsilateral difference waves. Following Sawaki and Luck’s (2010) methods, we measured the mean amplitudes of the target and target-like distractor difference waves between 225 ms and 300 ms to characterize the magnitudes of the N2pc components. Next, we measured the mean amplitude of the singleton-distractor difference wave in the same 115–225-ms interval used by Sawaki and Luck to characterize the magnitude of the P_D_. In addition, we measured the mean amplitude of the singleton-distractor difference wave in a later 225–300-ms interval that maps onto the timing of the P_D_ in studies from our lab (e.g., 250–290 ms in Gaspar & McDonald, 2014, and Gaspar et al., 2016; 220–260 ms in Hickey et al., 2009). To determine if a component of interest was present, the mean amplitude was tested against zero microvolts in a one-tailed, one-sample* t* test (see also Christie et al., 2025). We decided a priori to perform one-tailed tests for two reasons. First, each of the display configurations of interest was expected to elicit either an N2pc (target and target-like distractor displays) or a P_D_ (singleton-distractor display), but not both. Second, by definition, the N2pc and P_D_ are negative and positive voltages, respectively. Significant voltage differences in unexpected directions would not have been interpretable given Sawaki and Luck’s (2010) findings, thereby making one-tailed tests appropriate. Comparisons of N2pc or P_D_ amplitudes were done using two-tailed, paired-samples *t* tests.

The onset latencies of the N2pc and P_D_ components were assessed in two ways. First, we measured the time at which the components first reached 30% of their peak amplitudes. These measures were taken from jackknife (*N* − 1) sub-averages rather than the individual-participant difference waveforms to ensure that each waveform would contain the component of interest (latencies of missing components are undefined). The obtained *t* values were adjusted using the conventional jackknife method because of reduced variability in the subaverages (i.e., dividing the *t* value by *N* − 1; Miller et al., 1998, 2009). Latencies were compared using two-tailed tests to enable interpretation of differences in unexpected directions (e.g., nontarget N2pc beginning before target N2pc). Second, we plotted 95% confidence intervals around the contralateral-ipsilateral difference waves using the participant data used to create the grand-averaged difference waves. The time at which the confidence intervals were first fully negative (for N2pc) or fully positive (for P_D_) was taken as a second measure of onset latency. Confidence intervals had to be fully negative or fully positive for a minimum of ten consecutive sample points to avoid reporting the timing of spurious peaks.

### Results and discussion

#### Behavior

The mean hit rate for targets was 60.2%. The mean false-positive rate was 26.3% for target-like distractors, 5.6% for singleton distractors, and 6.0% for standard arrays. For comparison, in Sawaki and Luck’s (2010) second experiment, the hit rate for targets was 71.6% (11.4% higher), and the false-positive rate for target-like distractors was 14.8% (11.5% lower). Thus, it appears that it was more difficult to distinguish between targets and target-like distractors in the present experiment than in Sawaki and Luck’s (2010) experiment. Their false-positive rate for singleton distractors (5.0%) was about the same as in the present study. This indicates that the irrelevant singleton was no more or less distracting in the present study than in their study. The mean RT for targets in the present experiment (517 ms) was similar to that in Sawaki and Luck’s second experiment (534 ms).

#### ERPs

**Fig. 2:**
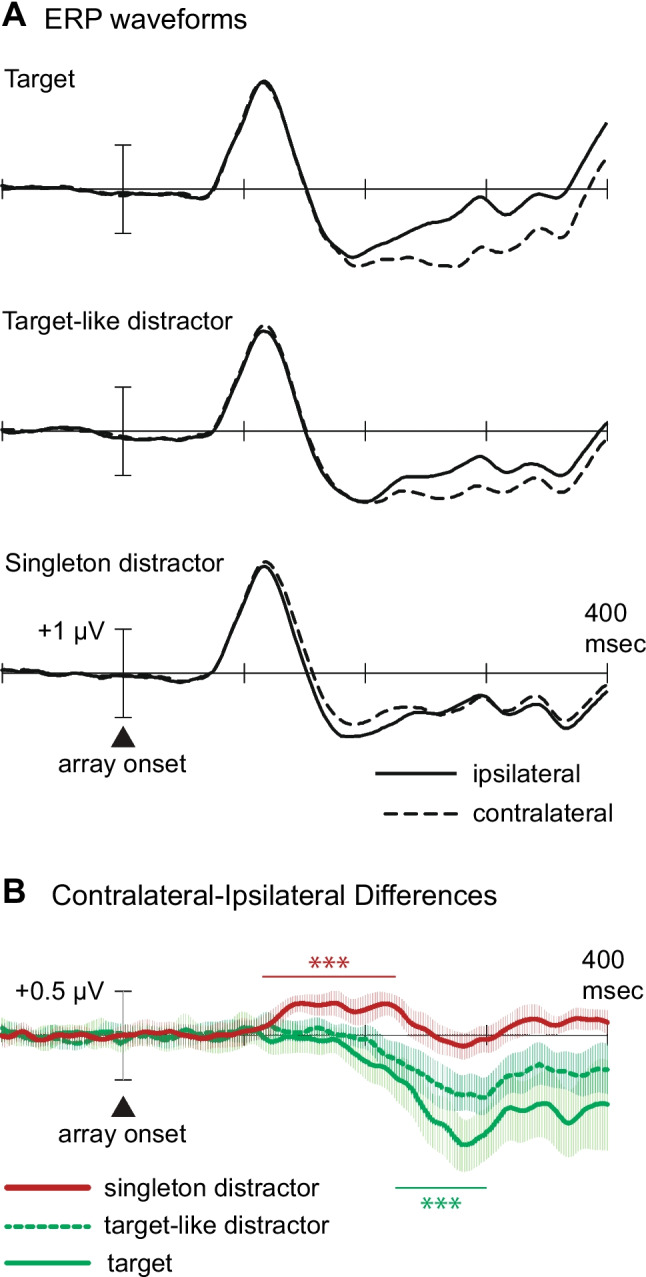
Grand-average ERPs from lateral occipital electrodes (PO7/PO8) in Experiment 1. **A** ERP waveforms recorded contralateral and ipsilateral to the target, target-like distractor, and salient distractor. **B** Contralateral-minus-ipsilateral difference waves corresponding to the lateral-occipital ERPs. Waveforms are plotted positive up, so the N2pc and P_D_ appear as downward and upward deflections, respectively. Error bars reflect 95% confidence intervals around the difference waveforms. Measurement windows are depicted by the horizontal lines above or below the *x*-axis. ***p <.001. (Color figure online)

Figure 2 displays the ERPs recorded over the lateral occipital scalp (at electrodes PO7 and PO8), grand-averaged across all participants. Waveforms recorded contralateral and ipsilateral to targets and target-like distractors largely overlapped in the time range of the first positive peak (P1; 90–130 ms), but the contralateral waveform became more negative than the ipsilateral waveform approximately 180–200 ms after onset of the letter arrays. These contralateral negativities indicate that the target and target-like distractor both elicited the N2pc component, as they did in Sawaki and Luck’s (2010) full-field letter-search task. Statistical analyses confirmed the presence of both the target N2pc (*M* = −0.93 µV), *t*(26) = −7.01, *p* <.001, *d*_z_* = −*1.35, and the target-similar-distractor N2pc (*M* = −0.52 µV), *t*(26) = −5.20, *p* <.001, *d*_z_ *= *−1.00. As in Sawaki and Luck’s study, the target N2pc was significantly larger than the target-like-distractor N2pc, *t*(26) = −4.46, *p* <.001, *d*_z_* =* −0.86. The 30% fractional peak latencies for the target N2pc and target-like distractor N2pc did not differ statistically (215 ms vs. 216 ms), *t*(26) < 1, but the first fully negative 95% confidence interval occurred earlier for the target N2pc (188 ms) than for the target-like distractor N2pc (214 ms). This latter difference indicates that the target N2pc began earlier than the nontarget distractor N2pc.

The occipital ERP waveforms recorded contralateral and ipsilateral to the salient distractor revealed a contralateral positivity that began approximately 120 ms poststimulus (onset latency: 118 ms; first fully positive confidence interval: 124 ms) and lasted until approximately 235 ms poststimulus (last fully positive confidence interval: 234 ms). Statistical analysis confirmed the presence of this contralateral positivity in the 115–225-ms measurement window used by Sawaki and Luck (2010) (*M* = 0.30 µV), *t*(26) = 7.63, *p* <.001, *d*_z_ *=* 1.47. The contralateral positivity was not significant in the 225–300-ms window (*M* = 0.03 µV), t(26) < 1.

Overall, the results of Experiment 1 replicated those of Sawaki and Luck’s (2010) full-field letter-search task. Targets and target-like distractors both elicited the N2pc component, which indicates that participants attended to both types of stimuli. Singleton distractors did not elicit the N2pc component, which indicates that participants did not attend to the salient-but-irrelevant color singletons in the same way as they attended to target-like distractors. Instead, singleton distractors were found to elicit a contralateral positivity that began approximately 100 ms before the onset of the target N2pc (onset latencies 118 ms vs. 215 ms), *t*(26) = 9.1, *p* <.001. The remaining question is whether this contralateral positivity reflected suppression of a singleton-driven priority signal, as asserted by Sawaki and Luck, or was associated with the priority signal itself. We addressed this question directly in Experiment 2.

## Experiment 2

The purpose of Experiment 2 was to determine whether the color singleton from Experiment 1 would continue to elicit an early contralateral positivity if participants were instructed to detect the singleton rather than one of the standard-color letters. Such a “target positivity” would be more difficult to measure because salient singleton targets elicit N2pc components starting as early as 160 ms (e.g., Brisson et al., 2007; Gaspar & McDonald, 2014). Given its larger magnitude, a target N2pc would override any target positivity after ~160 ms. Thus, we set out to measure a contralateral positivity in a shorter time window preceding the target N2pc, from 115 ms to 160 ms. Additionally, we obtained ERPs separately for upper- and lower-field targets, because the N2pc is smaller for targets in the upper field than for targets in the lower field (Luck et al., 1997; Tay et al., 2019). We reasoned that, while the N2pc to lower-field targets might completely obscure an early contralateral positivity, the N2pc to upper-field targets might be small enough to enable measurement of an early contralateral positivity like the one observed in Experiment 1 of the present study (and discovered by Sawaki & Luck, 2010). If the early contralateral positivity reflects suppression that prevents attentional processing of the salient distractors, it should not be elicited by the singleton target in Experiment 2.

### Methods

Thirty-three different students from Simon Fraser University participated in Experiment 2. Data from six participants were discarded due to excessive ocular artifacts. Of the remaining 27 participants (mean age: 19.0 years), 23 were women and 24 were right-handed. All participants reported normal or corrected-to-normal visual acuity and were tested for normal color vision using Ishihara color plates. No participant reported a history of neurological disorder.

Experiment 2 was identical to Experiment 1, except that the color singleton served as the target in all blocks. The identity of the singleton letter varied randomly across trials, as it did in Experiment 1. Participants were instructed to press the response button as quickly as possible whenever a letter array contained a different-colored letter (i.e., a color singleton). As such, there were now only two array types: Standard arrays, which contained eight same-color letters (1,440 trials; ~71.4%), and singleton-target arrays, which contained seven same-color letters and one color singleton (576 trials; ~28.6%). As before, an equal number of small letters and large letters appeared in each rectangle, and the colors of the standard letters and singleton letter were swapped randomly across blocks.

Due to the increased salience of the target in Experiment 2, the target N2pc was expected to occur earlier than it did in Experiment 1. To determine an appropriate time window within which to measure the mean amplitude of this target N2pc, we obtained the grand-averaged peak negativity within a 100–300-ms window, determined the time at which the N2pc first reached 30% of this peak negativity, and then used this latency as the lower bound of a 75-ms mean-amplitude measurement window (i.e., 178–250 ms). The magnitude of the early contralateral positivity was quantified as the mean amplitude in a 115–160-ms time window. One-tailed, one-sample *t* tests were used to test for the presence of each signed component.

All responses fell within the 100–800-ms response window, so it was not necessary to remove any trial due to an unacceptably fast or slow response. An average of 12.2% of trials were discarded from the ERP analyses because of artifact contamination.

### Results and discussion

The mean hit rate for detection of the singleton target was 97.7%. The mean false-positive rate for all other standard arrays was 0.3%. Across participants, the mean RT for targets was 414 ms. This RT was approximately 100 ms shorter than the mean RT for the nonsingleton letter targets in Experiment 1 (517 ms), *t*(52) = 7.93, *p* <.001, *d* = 2.16. These results indicate that it was easier to detect the singleton target in Experiment 2 than it was to detect the nonsingleton target in Experiment 1.

**Fig. 3:**
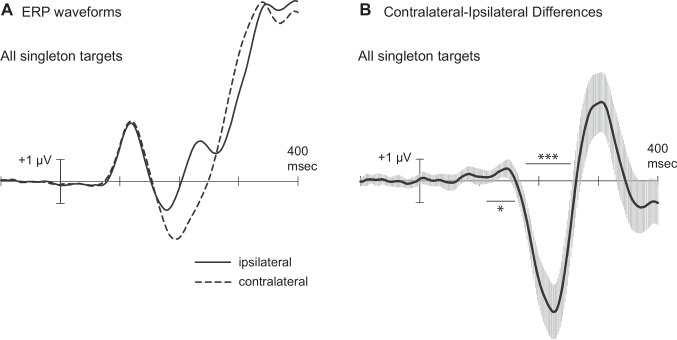
Grand-average ERPs to singleton targets in Experiment 2. **A** ERP waveforms recorded by contralateral and ipsilateral occipital electrodes (PO7/PO8). **B** Contralateral-minus-ipsilateral difference waves with 95% confidence intervals. Measurement windows are depicted by the horizontal lines above or below the *x*-axis *p <.05. ***p <.001

**Fig. 4:**
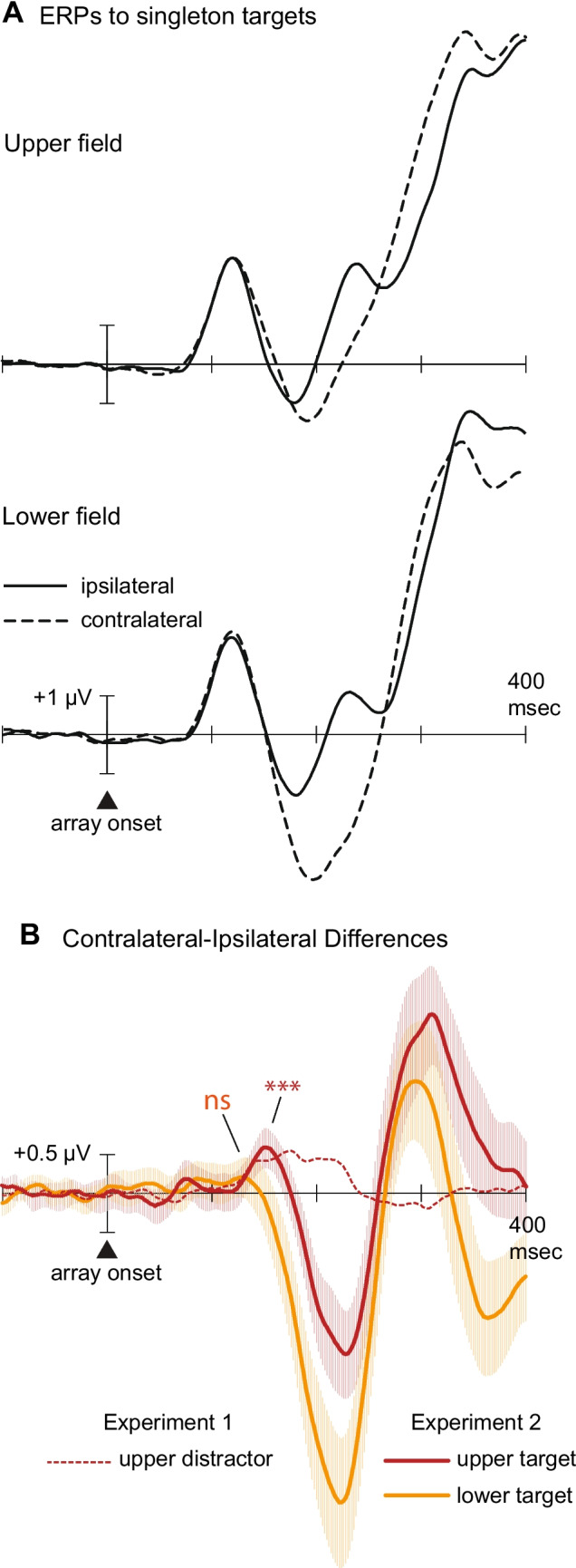
Experiment 2 ERPs plotted separately for upper- and lower-field targets. **A** ERP waveforms recorded by contralateral and ipsilateral occipital electrodes (PO7/PO8). **B** Contralateral-minus-ipsilateral difference waves with 95% confidence intervals. The difference wave for upper-field singleton distractors (Experiment 1) is included for comparison with the difference wave for upper-field singleton targets (Experiment 2). ***p <.001. (Color figure online)

Figure 3 displays the grand-averaged occipital ERPs recorded contralateral and ipsilateral to the singleton target in Experiment 2 (left), along with the corresponding contralateral-ipsilateral difference waves (right). Figure 4 shows the same waveforms and difference waves, separately for upper-field targets and lower-field targets. Contralateral and ipsilateral waveforms contained a P1 peak at 118–120 ms and a large P3 peak at 375–400 ms. As expected, the contralateral waveform was more negative than the ipsilateral waveform in the time range of the N2pc, from approximately 170 ms until approximately 250 ms poststimulus. Statistical analysis confirmed that the N2pc was present in a 178–253-ms measurement window for both upper-field targets (*M* = −1.40 µV), *t*(26) = −6.26, *p* <.001, *d*_z_* =* −1.21, and for lower-field targets (*M* = −3.09 µV), *t*(26) = −9.03, *p* <.001, *d*_z_ = −1.74. Notably, however, the N2pc was significantly smaller for upper-field targets than for lower-field targets, *t*(26) = 5.76, *p* <.001, *d*_z_* =* 1.11, and significantly later for upper-field targets (186 ms) than for lower-field targets (173 ms), *t*(26) = 2.69, *p* =.012.

We hypothesized that reducing and delaying the N2pc (by looking separately at upper-field targets) would enable better isolation of a small, temporally overlapping contralateral positivity. The results were in line with this component-overlap hypothesis. An early contralateral positivity was found for upper-field targets (*M* = 0.31 µV), *t*(26) = 3.20, *p* =.002, *d*_z_* =*.62, but not for lower-field targets (*M* = 0.04 µV), *t*(26) = 0.28, *p* =.39, *d*_z_ =.05. As shown in Fig. 4B, the mean amplitude of the upper-field “target positivity” did not differ from that of the upper-field “distractor positivity” in Experiment 1 (*M* = 0.38 µV), *t*(52) = 0.65, *p* =.519. Based on these findings, we conclude that the singleton targets elicited an early contralateral positivity as well as a temporally overlapping N2pc that partially or fully obscured measurement of the early contralateral positivity depending on whether the target was in the upper or lower field. This early contralateral positivity cannot reflect suppression, because participants rapidly attended to the singleton target (as evidenced by the N2pc, which peaked at 225–275 ms).

## General discussion

Sawaki and Luck (2010) hypothesized that salient visual stimuli trigger bottom-up priority signals that reflexively pull attention to their locations unless a top-down suppression process intervenes in time to prevent that from happening. In support of this signal-suppression hypothesis, they reported that a color singleton distractor elicits a relatively early P_D_ rather than an N2pc when participants search for a nonsingleton target. No P_D_ was observed in a control experiment, when participants closely monitored a narrow region around fixation. The disappearance of this positivity indicated that it was sensitive to the spatial distribution of attention and was not merely the result of an obligatory sensory process. However, other nonobligatory processes may have contributed to Sawaki and Luck’s P_D_. Here, we investigated whether their distractor-elicited positivity may have been associated with a singleton-driven priority signal rather than the suppression of a priority signal.

The present study sought to determine whether Sawaki and Luck’s (2010) distractor-elicited positivity was associated with a salience-driven priority signal or suppression of such a priority signal. We did this by measuring lateralized ERP activity associated with a color singleton when it was a distractor (Experiment 1) and when it was the target (Experiment 2). If the singleton-elicited positivity in Sawaki and Luck’s study was associated with a salience-driven priority signal, it should be evident when the singleton is a target or a distractor. By contrast, if the positivity was associated with suppression of that priority signal, it should be evident only when the singleton is a distractor.

Our alternative, priority-signal interpretation may seem at odds with the results of Sawaki and Luck’s (2010) control experiment if one assumes that the singleton-driven priority signal is triggered automatically. According to some researchers, however, preattentive computations of visual salience take place only within the currently attended region of space (the so-called “attentional window”; Belopolsky et al., 2007, Belopolsky & Theeuwes, 2010; Theeuwes, 2010). By this account, bottom-up salience computations would have been confined to the region of the fixation search array in the control experiment. This possibility was built into the signal suppression hypothesis from the outset. Specifically, Sawaki and Luck asserted that the visual system automatically detects salient visual singletons unless spatial attention is strongly focused on a different region of the display. Thus, according to the signal suppression hypothesis, a peripheral color singleton would not be expected to trigger a salience-driven priority signal when attention is narrowly focused at fixation, as it was in Sawaki and Luck’s control experiment.

The singleton target in Experiment 2 was expected to elicit an N2pc starting approximately 160 ms after array onset (Gaspar & McDonald, 2014, Experiment 3; Gaspelin & Luck, 2018, Experiment 3). Such an N2pc would partially obscure a target positivity in the time range of Sawaki and Luck’s (2010) P_D_. To mitigate this potential component-overlap problem, we analyzed ERPs separately for upper- and lower-field singleton targets, on the grounds that the N2pc is known to be smaller for upper-field targets than for lower-field targets (Luck et al., 1997). Critically, upper-field singleton targets were found to elicit a contralateral positivity in the 115–160 ms measurement window, and the mean amplitude of this “target positivity” did not differ from the mean amplitude of the “distractor positivity” obtained in Experiment 1 (0.31 μV vs. 0.38 μV), *t*(52) = 0.65, *p* =.52, *BF*_01_ = 3.06. Based on these findings, we conclude that the contralateral positivities observed in the present study and in Sawaki and Luck’s seminal study are associated not with suppression but with singleton-driven priority signaling, at least in the pre-N2pc time interval (~100–200 ms).

Our results and conclusions have important implications for multiple theories of visual selection. If the most salient item in the attentional window invariably captures attention, as hypothesized by salience-driven-selection theory (Theeuwes, 2010), the singleton distractor should have elicited an N2pc (Hickey et al., 2006). If that item triggers a priority signal that must be suppressed to prevent distraction, as hypothesized by signal suppression theory, the singleton distractor (but not the singleton target) should have elicited a P_D_. Contrary to both theories, the singleton distractor in Experiment 1 elicited neither an N2pc nor a genuine P_D_ (since the early positivity was also elicited by the singleton target in Experiment 2). Thus, our results do not support either of these theories. By contrast, the results are consistent with the contingent capture theory (Folk & Remington, 1998; Folk et al., 1992) and the dimension-weighting account (Found & Müller, 1996; Liesefeld & Müller, 2019), according to which selection may be achieved by upweighting relevant features or featural dimensions.

The early “target positivity” in Experiment 2 is consistent with ERP results obtained in search tasks that involve competition between concurrent target and distractor singletons (e.g., Gaspar & McDonald, 2014; Jannati et al., 2013; McDonald et al., 2023; Oxner et al., 2024a, b). In this paradigm, lateralized ERP activity associated with one lateral singleton is isolated by placing the other singleton directly above or below fixation), where it will not trigger lateralized components such as the N2pc or P_D_ (Hickey et al., 2006, 2009; Woodman & Luck, 1999, 2003). Under such conditions, the lateralized singleton tends to elicit enlarged contralateral positive voltages in the time range of the P1 peak (100–200 ms), whether that singleton is a target or a distractor (Barras & Kerzel, 2016; Corriveau et al., 2012; Jannati et al., 2013; McDonald et al., 2023). Corriveau and colleagues (2012) referred to this activity as the *posterior contralateral* *positivity* (*Ppc*) and ascribed it to salience-related processes. The Ppc is followed immediately by an N2pc when the singleton is a target (as in Experiment 2 of the current study) and is often followed by a second positivity in the N2pc time range when the singleton is a distractor. The second positivity cannot be ascribed to sensory processes or salience-driven priority signaling because it is modulated by task instructions (Gaspar & McDonald, 2014; Jannati et al., 2013). Thus, that second contralateral positivity appears to be a P_D_ associated with suppression or some other distractor-tagging process (Gaspar & McDonald, 2014; Gaspar et al., 2016; McDonald et al., 2023; Smit et al., 2020).

Although the ERPs obtained in additional-singleton search tasks suggest that singleton distractors are sometimes suppressed, the overall pattern of results do not support the hypothesis that suppression is necessary to prevent distraction (i.e., the signal suppression hypothesis). In particular, we have found that a salient color singleton elicits a P_D_ in the N2pc time range when observers search for a less salient color singleton (i.e., with unidimensional competition) but not necessarily when they search for a shape singleton (i.e., with cross-dimensional competition; Gaspar & McDonald, 2014; Jannati et al., 2013). Furthermore, even with unidimensional competition, only distractors that are considerably more salient than the target elicit the P_D_ (Gaspar et al., 2016). These findings suggest that suppression may be applied to prevent salience-driven distraction in certain situations but that facilitatory processes, such as dimension-weighting, may help guide attention to the target in other situations (Gaspar & McDonald, 2014).

Although there was no clear evidence for distractor suppression in the present study, it is possible that the contralateral positivity elicited by the singleton distractor in Experiment 1 was composed of an early priority-related positivity (Ppc) and a later suppression-related positivity (P_D_). As shown in Figs. 2 and 3, the contralateral positivity began in the P1 time range (~115 ms) but extended beyond 200 ms, into the N2pc time range. Sawaki and Luck’s (2010) distractor-elicited positivity showed a similar sustained time course when target and distractor colors remained fixed within blocks (Experiments 1 and 2). Critically, however, when target and distractor colors swapped unpredictably within blocks (Experiment 4), Sawaki and Luck’s positivity terminated by 200 ms. This demonstrates that singleton distractors elicit an early contralateral positivity (herein interpreted as a Ppc) without a later phase of positivity that could reflect suppression (i.e., a P_D_) in some conditions. Such findings are at odds with the hypothesis that suppression is necessary to prevent salience-driven distraction.

There has been substantial interest and research on salience-driven distraction and signal suppression since Sawaki and Luck’s (2010) seminal study. ERP studies often look for the P_D_ in a wide time window that begins in the time range of the P1 (starting 100 ms poststimulus) and extends beyond the conventional N2pc time window (until 500 ms in some cases; for review, see Gaspelin et al., 2023). The potential pitfall of using such a wide window is that the component of interest (the P_D_) might be conflated with other positive ERP components occurring before or after it. Here, we highlight one case in which the P_D_ was apparently conflated with an earlier Ppc component. Previously, we demonstrated that a late “cue P_D_” that occurred 400–500 ms after a cue array was time-locked to a subsequent target array and appeared to reflect perceptual enhancement of the cued search item rather than suppression of the previous cue (Hoffmeister et al., 2022; Livingstone et al., 2017). Thus, while the timing of the P_D_ may be variable, care must be taken to avoid conflation with other posterior contralateral positivities that fall outside of the N2pc time range.

Finally, we recommend reversing task instructions whenever possible to determine whether a putative P_D_ is triggered exclusively by irrelevant stimuli or whether the positivity continues to be evident when the same stimulus is a target. Such a task reversal was critical to the discovery of the P_D_ (Hickey et al., 2009), but the method has been implemented infrequently ever since (e.g., Gaspar & McDonald, 2014; Gaspelin & Luck, 2018; Jannati et al., 2013). When determining whether a singleton target elicits an early contralateral positivity, one may need to take steps to mitigate potential problems due to overlap by the N2pc. Otherwise, a contralateral positivity may appear to be absent when it is partially or fully obscured by a temporally overlapping N2pc (as was the case for lower-field targets in Experiment 2).

## Data Availability

The EEG files are available upon request to the corresponding author. Processed data, including RTs, ERP component latencies, and ERP component amplitudes are available at: https://osf.io/xgc97/

## Footnotes

1 The dimension-weighting account proposes that the weights are attached to feature dimensions rather than specific features.

## Code availability

Computer-controlled experiments written and run in Presentation (Neurobehavioral Systems, Inc) are available upon request to the corresponding author. All analysis scripts are available upon request to the corresponding author.
